# A rapid educational intervention to enhance brain MRI interpretation skills of radiology professionals for dementia diagnosis in Uganda: a pre- and post-intervention study

**DOI:** 10.1186/s12909-026-08997-z

**Published:** 2026-03-12

**Authors:** Rita Nassanga, Kawooya Grace Michael, Noeline Nakasujja, Martha Sajatovic, Stephen E. Jones, Mark Kaddumukasa

**Affiliations:** 1 Department of Radiology, College of Health Sciences, Makerere University, Kampala, Uganda; 2 Ernest Cook University, Kampala, Uganda; 3 Department of Psychiatry, College of Health Sciences, Makerere University, Kampala, Uganda; 4 University Hospitals Cleveland Medical Center, Case Western Reserve University School of Medicine, Cleveland, OH USA; 5 Imaging Institute, Cleveland Clinic Lerner College of Medicine, Cleveland, OH USA; 6 Department of Medicine, College of Health Sciences, Makerere University, Kampala, Uganda; 7 Department of Radiology, School of Medicine, Makerere University, Kampala, Uganda

**Keywords:** Dementia, Brain MRI, Imaging personnel, Training

## Abstract

**Introduction:**

Accurate interpretation of brain magnetic resonance imaging (MRI) data is vital for diagnosing dementia, as it can guide physicians toward proper diagnosis and management. In low-income settings such as Uganda, the challenges are greater due to limited diagnostic resources and gaps in radiologists’ skills. Brain MRI is rarely used in dementia diagnosis, and even when it is employed, subtle but critical findings, such as mild regional atrophy, are often overlooked. This highlights the pressing need to enhance the MRI interpretation skills of imaging practitioners, ensuring that essential diagnostic clues are not missed in patients with suspected dementia.

**Objective:**

To determine the effect of a training intervention on the brain MRI interpretation skills of radiologists and radiology residents among patients with a clinical diagnosis of dementia.

**Methods:**

We conducted a pre- and post-test study among practising radiologists and radiology residents. Participants completed a baseline written test on dementia-related MRI concepts and an image-interpretation test, attended a one-day, in-person training workshop led by expert radiologists and neurologists, and repeated the same assessments four weeks later. Complete paired data were available for 29 of 31 attendees. Wilcoxon signed-rank tests were used to compare pre- and post-training scores within groups, and Mann-Whitney U tests were used to compare change scores between cadres and MRI experience levels.

**Results:**

The median theoretical knowledge score increased from 73.3% (IQR 66.7–86.7) at baseline to 86.7% (80.0-93.3) after training (*p* < 0.001). Median image-interpretation scores improved from 50.9% (40.9–68.2) to 83.6% (73.6–89.1) across all participants (*p* < 0.001). Both radiologists and residents demonstrated significant gains, with residents showing slightly larger median improvements. Although both MRI experience groups (< 3 and ≥ 3 years) showed significant pre-post gains, participants with < 3 years of MRI exposure achieved a greater median increase in image-interpretation scores (+ 14.75 vs. + 12.0; *p* = 0.037).

**Conclusion:**

A focused, one-day training workshop substantially improved radiologists’ and residents’ knowledge and brain MRI interpretation skills for dementia in a low-resource setting, with particularly large gains among those with less MRI experience. These findings support integrating short, structured brain MRI dementia modules into residency and CPD programmes, although larger studies with longer follow-up are needed to confirm the durability and clinical impact of these improvements.

**Supplementary Information:**

The online version contains supplementary material available at 10.1186/s12909-026-08997-z.

## Introduction

The global prevalence of dementia is rapidly increasing, with estimates suggesting that it will double every 20 years and that a significant burden will fall on low- and middle-income countries (LMICs) [1–4]. Dementia, which is caused primarily by Alzheimer’s disease, affects cognitive and behavioral functions, disrupting daily activities [5–7]. It affects 7–8% of individuals over 65 years old and 30% of those over 80 years old [7]. Early and accurate diagnosis is crucial for managing symptoms and improving quality of life. A careful history and neurological examination, along with biomarkers from blood, cerebrospinal fluid, and imaging, are essential for diagnosing dementia. Among these, brain MRI serves as a key diagnostic, non-invasive tool due to its high spatial resolution, which allows for the detection of subtle morphological changes indicative of the condition [6, 8, 9].

In Uganda, where this study was conducted, the increase in life expectancy from 39.0 years in 1951 to 63.4 years in 2020 has led to an increase in age-related conditions such as dementia, creating a significant socioeconomic burden and impacting patients’ quality of life [10–13]. A population-based study in rural Uganda reported that nearly 20% of older adults screened positive for dementia [14]. Another study reported that 13.2% of elderly patients admitted to non-psychiatric wards had dementia, making it one of the most common diseases among elderly individuals [14]. Despite the growing prevalence of dementia, there is a notable gap in standard radiology training concerning dementia evaluation. Many imaging centers in low-resource settings lack standardized protocols for brain MRI, and radiologists often overlook subtle atrophy patterns crucial for accurate diagnosis [15, 16]. This highlights a critical need for enhanced training in interpreting brain MR images for dementia patients.

Compared with developed countries, where brain MRI is routinely used to assist with early dementia diagnosis, low-income settings face significant challenges in integrating this imaging modality into clinical practice. The American Academy of Neurology, American College of Radiology Appropriateness Criteria, and Ontario Neurodegenerative Disease Research Initiative recommend anatomical neuroimaging for dementia patients to identify the pathological cause of dementia syndrome [17, 18]. In high-resource countries, brain MRI is a cornerstone of dementia diagnosis, revealing treatable conditions and detecting region-specific atrophy patterns characteristic of primary dementia disorders [19, 20]. MRI is also excellent at evaluating cortical atrophy, microbleeds, lesions that restrict diffusion, and subtle vascular lesions [21].

The appropriate radiological diagnosis of dementia involves using the right protocol for MRI sequences as well as making a standardized report for the images. The use of visual rating scales helps the reviewer to assess and quantify the structural and volume changes systematically and thus report the findings in a standardized way [22]. The visual rating scales enable reproducible semiquantitative assessment of volume loss [23, 24] and reduce interrater variability [24]; however, they have a subjective element, and their application is dependent upon appropriate training and prior experience of the radiologist.

In Uganda, the diagnostic landscape for dementia faces significant challenges. Limited healthcare infrastructure, low community awareness, and a shortage of specialists, such as neurologists and neuropsychologists, contribute to delayed diagnoses, especially in rural areas. Many healthcare professionals lack mental health training, and neuropsychological tests are rarely administered [25]. Even when brain MRI data are available, subtle findings such as regional atrophy are often missed because of insufficient specialized training among radiologists. The absence of standardized MRI protocols and reporting systems further complicates accurate diagnosis. Training curricula offer minimal content on dementia-specific MRI interpretation, and there are few continuous professional development opportunities to build competency. High MRI costs, limited access, and scarce interdisciplinary collaboration with neurologists and psychiatrists further hinder timely diagnosis [26, 27]. Addressing these gaps through targeted educational interventions and improving collaboration between radiologists and other specialists is crucial. Therefore, in Uganda, despite the availability of brain MRI in some centres, radiologists and residents often lack the training to identify subtle but clinically relevant imaging features of dementia, leading to underdiagnosis and mismanagement. This study, therefore, aimed to assess the impact of a tailored educational program designed to enhance the brain MRI interpretation skills of radiology professionals in dementia diagnosis.

## Methods

### Study design

This was an interventional study with a pre-test and posttest conducted among radiologists and radiology residents.

### Study setting and mode of delivery

The training was conducted as an in-person, one-day workshop held at the Department of Radiology, Makerere University College of Health Sciences in Kampala, Uganda. All participants attended physically in a seminar room equipped with a projector for didactic lectures and workstations for case-based image review and group discussions.

### Participants and sampling

This study involved 31 participants with knowledge of the imaging and diagnosis of dementia through the interpretation of brain MR images. The participants included 17 radiologists and 14 radiology residents from six different health facilities selected purposively. Eligibility included having participated in writing brain MRI reports in the previous three months and providing informed consent for participation in the study.

### Study procedures and assessments

The training workshop was delivered in person; however, both the pre-test and post-test assessments were conducted remotely. For each assessment, participants were emailed high-resolution soft-copy PowerPoint (PPT) files containing the brain MRI cases and corresponding questions. Participants reviewed the cases on their own computers and recorded their answers on structured response sheets, which they returned to the study team via email. No printed films were used, and the assessments were completed outside the formal PACS reporting environment, in a format similar to an asynchronous case-based quiz.

### Pre-training assessments

Two pre-tests were administered to establish participants’ baseline knowledge and skills in brain MRI interpretation for suspected dementia.

#### Pre-test I: theoretical knowledge assessment

This assessment targeted theoretical knowledge and comprised 15 objective, single-choice questions with four possible answers, aimed at assessing participants’ understanding of MRI sequences and the specific/signature imaging findings associated with various types of dementia. The content covered typical and atypical dementia subtypes, including Alzheimer’s disease, vascular dementia, frontotemporal dementia, dementia with Lewy bodies, and rarer forms such as multisystem atrophy (both the cerebellar and putaminal subtypes), progressive supranuclear palsy, corticobasal degeneration, Creutzfeldt-Jakob disease, and Huntington’s disease. Each question had one best answer. Each correct response was awarded one point, giving a maximum total score of 15 points.

#### Pre-test II: image interpretation assessment

This assessment targeted skills in the interpretation of brain MRI scans from 11 clinical cases of patients with suspected dementia. The participants were required to identify pertinent MRI findings and differentiate between the various dementia subtypes. The cases presented a mix of typical and atypical imaging features representing the spectrum of dementia disorders outlined in Pre-Test I. For each case, participants were tasked with identifying the MRI sequences (1 point), describing the relevant brain MRI findings (2 points), and providing the final or most likely diagnosis (2 points), resulting in a maximum of 5 points per case and 55 points in total. For the 2-point description and diagnosis components, we allowed partial credit **(**1 point) when responses were partially correct but did not fully meet the predefined criteria.

The allocation of points and the scoring rubric were developed a priori by a panel of two neuroradiologists and one neurologist, based on content relevance and typical weighting in dementia MRI interpretation, and piloted on two non-participant radiologists to ensure clarity.

### Training program

Following the baseline assessments, all participants attended a one-day, in-person interactive training workshop on brain MRI interpretation in dementia. The workshop lasted approximately 7.5 h (full-day session), delivered across morning, mid-morning, and afternoon sessions. The training was facilitated by neurologists and radiologists and covered: an introduction to dementia; clinical features; standard brain MRI protocols; visual rating scales; and characteristic imaging features of typical and atypical dementia subtypes. As part of the workshop, participants were also introduced to a structured brain MRI reporting template for dementia, and example cases were discussed using this format. Training methods included didactic lectures, case-based presentations, question-and-answer sessions, and interactive group discussions.

Eleven MRI cases were selected for the image-interpretation assessment. Six cases were anonymised clinical scans retrieved from the PACS archive of our university-affiliated tertiary hospital, and five were high-quality teaching cases sourced from publicly available radiology education platforms, including RSNA Radiographics. All locally sourced cases were fully anonymised in compliance with institutional ethics approval. Each case was independently reviewed and verified by two consultant radiologists with more than 10 years of experience in neuroradiology to confirm the accuracy of the imaging findings and diagnoses. The selection intentionally represented a spectrum of common and atypical dementia patterns, including Alzheimer’s disease, vascular dementia, frontotemporal dementia, dementia with Lewy bodies, Creutzfeldt-Jakob disease, Huntington’s disease, and atypical parkinsonian syndromes.

In line with the CONSORT (Consolidated Standards of Reporting Trials) extension and the TIDieR (Template for Intervention Description and Replication) guidance, a structured description of the educational intervention, including trainers, delivery methods, materials, and duration is presented in Supplementary File 1, whereas Table 1 summarises the curriculum content.

**Table 1:** Summary of the training curriculum, outlining the topics, core content, and key concepts on dementia and brain MRI interpretation covered during the workshop

Topic	Content and key concepts
Introduction	Epidemiology of dementia classification of dementia
Clinical aspects of dementia	Risk factors and definition of dementia Types of dementias Diagnosis and Investigations
Role of MRI in dementia	Rule out other causes of cognitive impairment. Identify early onset AD for possible innovative therapy and counselling
MRI Techniques	Image acquisition Protocols Sequences
Visual rating scales in brain MRI	Medial Temporal Atrophy(MTA) scale Global Cortical Atrophy(GCA) scale Frontal atrophy scale Koedam score Fazeka scale
Changes on brain MRI	Structural changes Volume changes
Typical imaging patterns of common dementia types	Alzheimer’s dementia Frontotemporal dementia Vascular dementia Dementia with Lewy bodies
Typical imaging patterns of less common dementia types	Corticobasal degeneration Progressive supranuclear palsy Multisystem atrophy Huntington’s dementia Creutzfeldt Jakob disease
Post training quiz	Case reviews for various dementia types
Report Template for brain MRI in dementia	Sections for writing a standard brain MRI report template

### Post-training assessment

Four weeks after the workshop, participants completed a post-test that was identical in format and content to the pre-tests. The same PPT-based cases and structured answer sheets were emailed to participants, who completed the assessments remotely using their computers. This allowed direct comparison of pre- and post-training theoretical knowledge and image interpretation performance. The four-week interval was chosen to allow time for consolidation of learning and to minimise recall of specific pre-test items or training cases, providing a more realistic estimate of short-term retention.

### Scoring and gold standard development

Participants completed their answer sheets electronically and returned them via email. All answer sheets were anonymised before scoring and assessed independently by two senior consultant radiologists who were not involved in delivering the training.

A structured answer key (“gold standard”) was developed a priori by a panel consisting of two consultant radiologists and one neurologist. For each MRI case, the panel agreed on:

- key sequences expected,
- essential imaging features (atrophy, small-vessel disease, focal lesions, atypical patterns), and.
- the most likely dementia subtype diagnosis.

This formed the scoring rubric, used to grade responses for sequence identification (1 mark), imaging description (2 marks), and final diagnosis (2 marks). Partial credit (1/2 marks) was awarded when responses were partially correct but incomplete.

Scores from the two raters were compared, and any discrepancies were resolved through discussion to reach consensus. Final total scores were used for analysis.

### Post-training evaluation

At the conclusion of the training, the trainees completed a quiz to reinforce the key concepts covered during the sessions. They were introduced to a structured reporting template for the systematic interpretation of brain MR images in patients with suspected dementia. Table 2 summarizes the cases that were used in image interpretation during training.

**Table 2:** Brain MRI cases included in the image-interpretation assessment, illustrating typical and atypical dementia patterns with corresponding sequences, key findings, and final diagnoses

Number	Sequences	Findings	Diagnosis
1	T1W and T2FLAIR	Bilateral hippocampal atrophy, MTA grade IV	Alzheimer’s dementia
2	T2W	Left temporal and left inferior frontal gyral atrophy.	Frontotemporal dementia
3	T2FLAIR and T1W	Periventricular white matter hyperintensities; FAZEKA grade III and MTA atrophy grade IV	Vascular and Alzheimer’s dementia
4	SWI and T1W	Atrophy of the hippocampi. Multiple small foci of signal dropout on SWI.	Cerebral Amyloid Angiopathy
5	SWI	Reduced volume of the Putamen. Reduced signal of the putamen relative to globus pallidus on GRE/reversal of the expected magnetic susceptibility pattern within thethin theein themagglobus pallidus on GRE.	Multisystem atrophy-Parkinsonian type
6	T2FLAIR and DWI	Changes in the neocortex appearing hyperintense on FLAIR and restricting on DWI with a Cortical ribbon sign.	Creutzfelt Jakob dementia
7	DWI and T2FLAIR	The basal ganglia (Globus pallidus and caudate nuclei)and the posterior thalamus are hyperintense and restrict on DWI. ict on DWI. ntense and restrhyperintensity of the pulvinar and dorsomedial thalamic nuclei bilaterally on FLAIR and DWI	Creutzfelt Jakob variant
8	T1W	Bilateral caudate atrophy.	Huntington’s dementia
9	T1W and T2W	Mid brain atrophy, Mickey mouse/humming bird	Progressive Supranuclear palsy
10	T2FLAIR and T1W	T2 hyperintensity forms a cross on axial images in the pons, indicative of the ‘hot cross bun’ sign. Cerebellar atrophy	Multisystem atrophy-Cerebellar type
11	T2FLAIR	Asymmetric parietal cortical atrophy.	Corticobasal degeneration

### Data analysis

Data from the baseline and post-training assessments were analysed using STATA version 17.0 (Texas, USA). Categorical variables were summarised using frequencies and percentages, while continuous variables were presented as medians and interquartile ranges (IQRs). Theoretical knowledge and image interpretation score distributions were visualised using box plots.

Score distributions were assessed for normality using the Shapiro-Wilk test and visual inspection of histograms and Q-Q plots. Because the bounded test scores violated normality assumptions, we used non-parametric tests: Wilcoxon signed-rank tests for paired pre-/post-training comparisons and Mann-Whitney U tests for between-group comparisons of change scores.

For subgroup analysis by cadre, Year II and III residents were collapsed into a single ‘Resident’ group to allow valid statistical comparison with radiologists, given the small number of Year II residents (*n* = 4), which limited the power for Kruskal-Wallis testing. Between-group comparisons for age (< 35 vs. ≥ 35 years) and MRI experience (< 3 vs. ≥ 3 years) were also conducted using the Mann-Whitney U test.

All statistical tests were two-sided, and a significance level of *p* < 0.05 was used.

This study was reported in accordance with the STROBE (Strengthening the Reporting of Observational Studies in Epidemiology) guidelines. The completed checklist is provided as Supplementary File 2.

## Results

### Demographic characteristics of the study participants

The median (IQR) age of the participants was 34 (33, 39) years. Slightly more than half were female (18/31) and qualified radiologists (17/31). Participants had a median (IQR) of 2 (2, 4) years of MRI reporting experience (Table 3).

**Table 3:** Characteristics of the radiologists and residents who participated in the study

Variable	Categories	Frequency (*N* = 31)	Percentage (%)
Age (years)	Median (IQR)	34 (33, 39)	
Sex	Female	18	58.1
	Male	13	41.9
Cadre	Radiologist	17	54.8
	Residents	14	45.2
Years of experience with MRI (years)	Median (IQR)	2 (2, 4)	

### Theoretical knowledge assessment

Of the 31 participants who completed the baseline assessment, 29 (93.5%) completed both the pre- and post-training assessments and were included in the paired analysis.

**Fig. 1:**
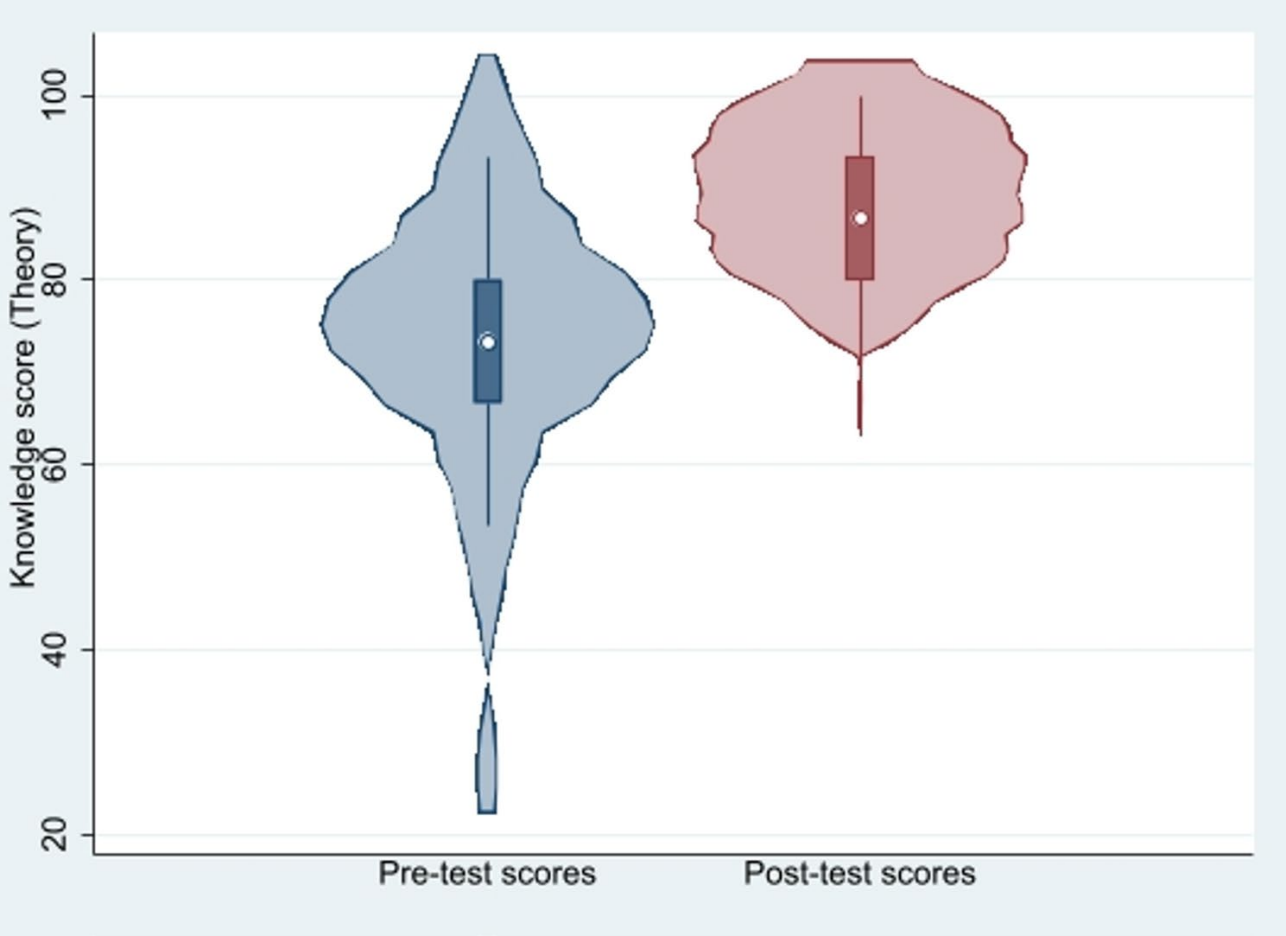
Distribution of theoretical knowledge pre- and post-test scores (*n* = 29 paired). Violin plot displays kernel density with embedded box plot (median and IQR). Pre- vs. post-test comparison: Wilcoxon signed-rank test, *p* < 0.001

The median (IQR) theoretical knowledge score increased from 73.3% (66.7, 80.0) at baseline to 86.7% (80.0, 93.3) after the educational intervention (Wilcoxon signed-rank test, *p* < 0.001) (Fig. 1).

### Subgroup changes in theoretical knowledge

Knowledge scores improved significantly after the intervention across sex and cadre subgroups (all *p* < 0.05). Participants with < 3 years of MRI experience demonstrated significant improvement, whereas those with ≥ 3 years of experience did not show a statistically significant change (Table 4).

**Table 4:** Sub-group analysis of change in knowledge scores before and after an educational intervention

Variable	Frequency *N* = 29 (%)	Pre-test knowledge core	Post-test knowledge score	*P* value
Sex				
Female	18(62.1)	73.3 (66.7, 80.0)	93.3 (86.7, 100.0)	0.001
Male	11 (37.9)	80.0 (73.3, 86.7)	86.7 (80.0, 93.3)	0.049
Cadre				
Radiologist	15 (51.7)	73.3 (73.3, 86.7)	86.7 (80.0, 93.3)	0.040
Resident	14 (48.3)	73.3 (60.0, 80.0)	93.3 (86.7, 100.0)	0.002
Years of experience				
<3 years	16 (55.2)	73.3 (60.0,80.0)	93.3 (86.7, 96.7)	0.001
≥3 years	14 (48.3)	80.0 (73.3, 86.7)	86.7 (80.0, 93.3)	0.106

### MRI image interpretation scores

**Fig. 2:**
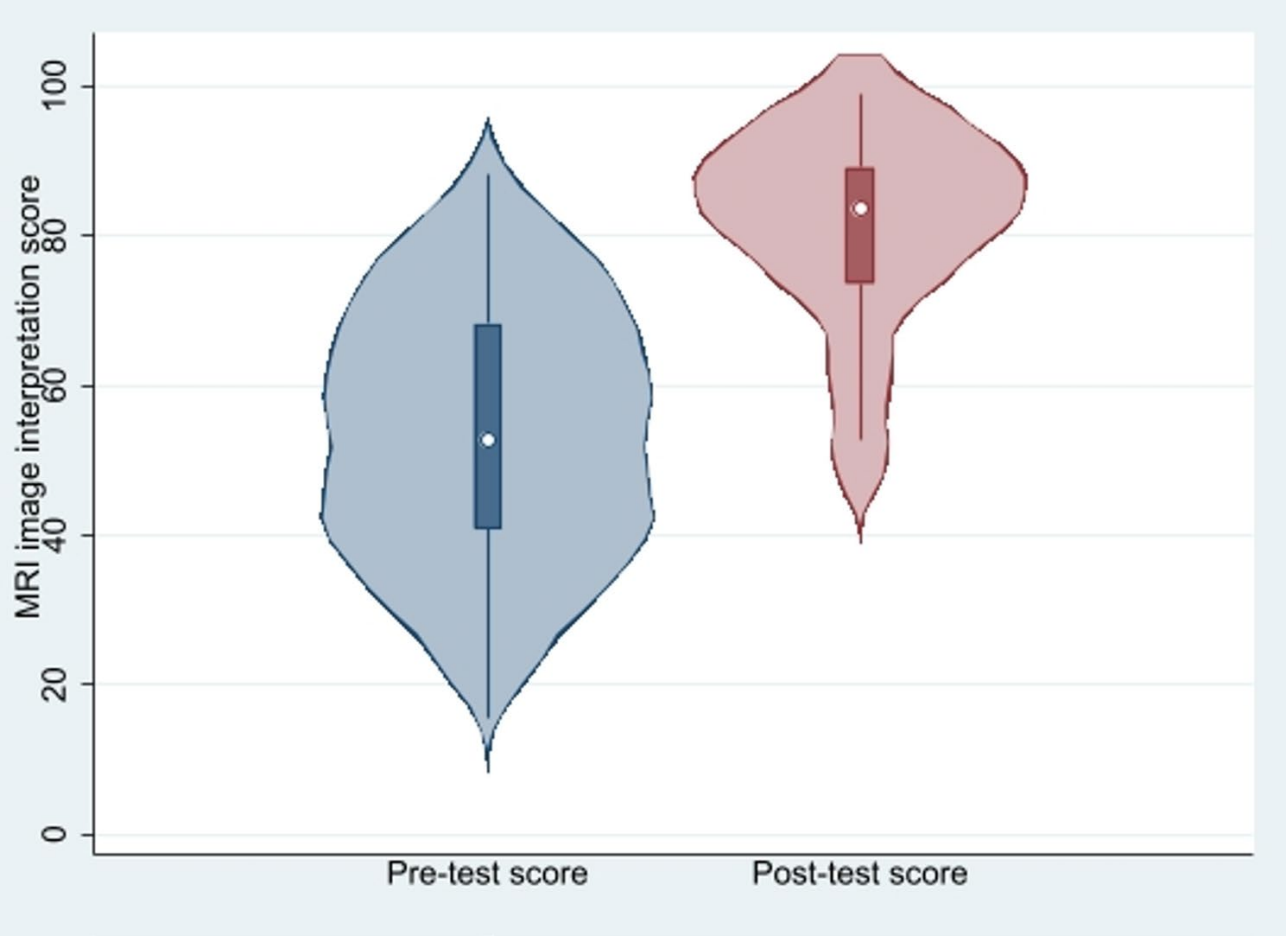
Distribution of image-interpretation pre- and post-test scores (*n* = 29 paired). Violin plot displays kernel density with embedded box plot (median and IQR). Pre- vs. post-test comparison: Wilcoxon signed-rank test, *p* < 0.001

The median (IQR) MRI image interpretation score increased from 52.7% (40.9, 68.2) before the intervention to 83.6% (73.6, 89.1) after the intervention (Wilcoxon signed-rank test, *p* < 0.001) (Fig. 2).

### Subgroup changes in MRI image interpretation

Image interpretation scores improved significantly across sex, cadre, and experience subgroups (all *p* < 0.05) (Table 5). Improvement was observed in both radiologists and residents, as well as in participants with < 3 years and ≥ 3 years of experience.

**Table 5:** Sub-group analysis of change in MRI image interpretation scores before and after an educational intervention

Variable	Frequency *N* = 29 (%)	Pre-test MRI Image reading score	Post-test MRI Image reading score	*P* value
Sex				
Female	18(62.1)	48.2 (34.5, 68.2)	78.2 (55.5, 89.1)	0.003
Male	11 (37.9)	54.6 (44.5, 70.0)	83.6 (80.0, 90.9)	< 0.001
Cadre				
Radiologist	15 (51.7)	49.1 (44.5, 73.6)	80.9 (66.4, 88.2)	0.001
Resident	14 (48.3)	55.5 (34.5, 63.6)	84.1 (80.0, 88.2)	0.001
Years of experience				
<3 years	16 (55.2)	55.5 (34.1, 64.6)	83.6 (78.2, 88.2)	< 0.001
≥3 years	14 (48.3)	49.1 (44.5, 73.6)	82.7 (69.1, 90.9)	0.002

## Discussion

The purpose of this study was to assess the effect of a targeted training program on the brain MRI interpretation skills of radiologists and radiology residents in relation to clinically suspected dementia. The results of this study demonstrate that an educational intervention can significantly enhance both theoretical knowledge and practical skills in brain MRI interpretation among radiology professionals, particularly in the context of dementia diagnosis. These findings are consistent with previous studies which highlighted that targeted educational programs have a positive impact on diagnostic accuracy and clinical performance in radiology, with well-designed radiology education projects enhancing radiologist training and patient care in low- and middle-income countries [28–30].

Given that MRI is a relatively new imaging modality in Uganda, imaging professionals have limited exposure compared with their counterparts in developed countries. Previous studies focusing on radiology training in Africa have shown that postgraduate radiology training programs in LMICs encounter several obstacles, such as insufficient medical equipment, inadequate standardization or implementation of training curricula, and a shortage of formally trained subspecialty instructors [26, 27]. When discussing radiology as a global service, Omofoye et al. suggested customizing training programs for the local environment to ensure the success of global radiology education projects [30]. Therefore, this study aimed to assess whether focused training could enhance imaging practitioner skills in interpreting brain MR images in dementia patients.

### Improvement in theoretical knowledge

The study findings revealed an improvement in theoretical knowledge. The significant increase in theoretical knowledge scores post-intervention underscores the value of structured educational training. This implies that the designed training intervention was effective in equipping the trainees with the requisite knowledge and skills to interpret brain MR images in patients with suspected dementia and calls for targeted training of radiologists in such specialized skills. Similar findings have been reported in other studies where radiology professionals showed enhanced understanding of complex imaging concepts following targeted training sessions. For example, a study by Salajegheh et al. demonstrated improved knowledge and skills in image interpretation in medical students after an e-learning course [28].

### Enhancement of practical skills

Improvements in radiology image interpretation skills among study participants are particularly noteworthy. Accurate interpretation of brain MR images is critical for the diagnosis of dementia, and our intervention successfully bridged the gap between theoretical knowledge and practical application. This outcome is supported by prior research, such as that of Sendra-Portero et al., who demonstrated that training in image interpretation significantly increased image interpretation skills among medical students [29]. This study adds to this body of evidence, showing that such improvements can be achieved across various subgroups, including those with differing levels of experience and training. A study in the USA reported that limited training in interpreting MRI may result in inadequate dementia assessment [31]. Other studies that have assessed interrater variability report that brain MRI changes can be challenging to identify in both research and clinical settings [32] thus training helps to bridge such gaps in skills.

While hippocampal atrophy was emphasized during training as a hallmark of Alzheimer’s disease, emerging evidence suggests that other structural markers, particularly white-matter abnormalities and ventricular enlargement-may also appear in early disease stages, even before hippocampal loss becomes prominent. Recent longitudinal MRI studies demonstrate that accelerated white-matter atrophy and ventricular expansion are associated with earlier progression from normal cognition to mild cognitive impairment (MCI), supporting their value as early neurodegenerative markers alongside medial temporal atrophy [33].

### Subgroup analysis

The consistent improvement observed across different demographic subgroups, such as gender, age, and years of experience, highlights the universal applicability of the educational intervention. These findings resonate with those of previous studies [34–36], which reported that educational programs in radiology can be effective across diverse participant profiles, reinforcing the idea that such interventions are crucial in enhancing overall diagnostic capabilities in radiology. In this study, all participants had some prior experience with MRI interpretation, which likely contributed to their increase in knowledge and skills, as they were not starting from a baseline of no understanding. However, this prior experience may not have specifically included brain MRI interpretation in the context of dementia, which underscores the value of the focused training provided in this study. The study revealed that younger trainees showed greater improvement in knowledge and skills after training. This may be attributed to the relatively recent inclusion of MRI training in radiology curricula and the increased availability of MRI equipment in the country. As a result, younger radiologists are likely to have had more exposure to MRI in terms of the number of cases than their older counterparts are, making training more effective for them.

Descriptively, female participants showed larger gains than males, despite slightly lower baseline scores. These descriptive sex-based patterns do not imply statistical significance or causal differences but may reflect variations in recent academic exposure, engagement during the workshop, or receptiveness to structured training tools such as visual rating scales, standardised MRI reporting templates, and case-based learning. As this analysis was descriptive only and not formally tested for significance, these trends should be interpreted cautiously.

### Implications for practice

Radiology practitioners are expected to interpret brain MR images for patients with clinical suspicion of dementia. Radiologists and residents are likely to encounter brain MR images of patients from psychiatry and neurology clinics routinely; however, there are no guidelines or structured reports on how to interpret images in this patient group. The patterns of atrophy, vascular and structural changes and visual rating scales may guide image interpretation [24, 37, 38]. However, it is not certain if they are used in everyday clinical practice in Uganda, and this does not align with the use of imaging in subtyping dementia, which would help in the early diagnosis of dementia. Referring physicians increasingly rely on the anatomical and pathological information provided by radiologists; thus, imaging practitioners must be able to extract pertinent information from radiological images. The findings from this study suggest that incorporating regular training into radiology programs could be an effective strategy to improve diagnostic skills, particularly in specialized areas such as dementia. Given the increasing reliance on advanced imaging techniques for accurate diagnosis, ongoing education and training are essential for ensuring that radiology professionals are equipped with the necessary skills to interpret complex imaging studies accurately. There was a significant increase in both theoretical knowledge and image interpretation skills following the training, aligning with the established understanding that training enhances knowledge and skill acquisition. These findings highlight the need for radiology training programs to incorporate more content focused on brain MRI in dementia diagnosis. In addition, the results suggest the potential value of continuing professional development (CPD) programs that specifically target these skills, with the training model used in this study serving as a foundation.

The importance of designing training programs to update the skills of imaging personnel needs to be emphasized. This is particularly relevant for low-resource settings where MRI is being newly integrated into dementia diagnosis. The duration of training, prior experience and content required to achieve acceptable competency is a subject of debate in many training interventions [39, 40].

While the study provides foundational evidence that training can significantly improve MRI interpretation skills, several limitations should be acknowledged. First, the small sample size and absence of a control group limit the ability to make causal inferences and reduce the generalizability of the findings. Second, both pre- and post-training assessments were administered remotely and unsupervised. Although participants were instructed to complete them individually without consulting reference materials, the possibility of external assistance, especially in the post-test, cannot be entirely excluded. Third, the follow-up period was limited to four weeks, which only permits evaluation of short-term retention; the long-term sustainability of the acquired skills remains unknown. Although the primary purpose of this pilot was not longer-term follow-up, future work should include longitudinal assessment of trained radiologists and residents at multiple time points (for example, 6–12 months) to evaluate long-term knowledge retention and application in routine reporting. Such data would help refine the curriculum and inform broader scale-up of this training into formal CPD programmes and residency teaching.

A methodological strength, however, is that score distributions were formally assessed using the Shapiro-Wilk test, and appropriate non-parametric tests (Wilcoxon signed-rank and Mann-Whitney U) were used to minimise violations of statistical assumptions, given the bounded nature of test scores and small sample. Despite these limitations, the study contributes to the limited evidence on structured MRI training for dementia in low-resource settings and suggests that similar interventions could be integrated into continuing professional development (CPD) programs.

### Feasibility and potential for integration into CPD and residency training

This one-day training model appears potentially feasible for incorporation into existing CPD activities and residency teaching formats in Uganda and similar low-resource settings. It was delivered using locally available MRI infrastructure and anonymised clinical cases from routine practice, and facilitated by local radiology and neurology faculty. These features suggest the approach could be adapted to common CPD formats (e.g., short workshops or in-service sessions) and embedded within neuroradiology rotations or case-based teaching. However, this study evaluated preliminary effectiveness over a short follow-up period and did not formally assess implementation outcomes such as feasibility at scale, acceptability, cost-effectiveness, or sustained impact. Future studies should include these implementation measures alongside longer-term skill retention.

## Conclusion

This study shows that a focused, one-day educational intervention can substantially improve both theoretical knowledge and practical brain MRI interpretation skills for dementia among radiologists and radiology residents in a low-resource setting. Participants showed clear gains in recognising dementia-related imaging patterns and applying structured visual rating scales. Improvements were observed in both radiologists and residents, with slightly larger gains among those with fewer years of MRI reporting experience.

These findings support the integration of short, structured brain MRI dementia modules into residency curricula and continuing professional development activities, especially where formal neuroradiology training is limited. However, the results reflect short-term retention in a small, uncontrolled cohort, and the durability and clinical impact of these gains could not be assessed. Future multi-centre, longitudinal studies with larger samples and longer follow-up are needed to determine whether such training leads to sustained reporting quality, better diagnostic pathways for dementia, and improved patient outcomes.

## Supplementary Information

Supplementary Material 1.

Supplementary Material 2.

## Data Availability

The data that support the findings of this study are available from the corresponding author upon reasonable request.
