# Supplementary material for: A rapid educational intervention to enhance brain MRI interpretation skills of radiology professionals for dementia diagnosis in Uganda: a pre- and post-intervention study
Source: BMC Med Educ. 2026 Mar 12;26:642. doi: 10.1186/s12909-026-08997-z (PMC13094059; doi:10.1186/s12909-026-08997-z)
Supplement: Supplementary file 1 — Supplementary Material 1. [file 12909_2026_8997_MOESM1_ESM.docx]

**Supplementary file 1: Provides a structured description of the educational intervention according to CONSORT/TIDieR guidance**

| **Item / Component** | **Description** |
| --- | --- |
| **Name** | Brain MRI Dementia Interpretation Training Workshop |
| **Rationale** | To improve radiology professionals’ knowledge and skills in interpreting brain MRI for dementia diagnosis, given limited dementia-focused MRI training and under-recognition of subtle, clinically relevant imaging findings in Uganda. |
| **Recipients (Who)** | Radiology residents and practising radiologists who were actively involved in brain MRI reporting within the previous three months. |
| **Providers (By whom)** | One consultant radiologist and one neurologist with expertise in dementia and neuroimaging. |
| **Setting (Where)** | In-person workshop held in a seminar room at Makerere University College of Health Sciences. |
| **Mode of delivery (How)** | Face-to-face, group-based teaching using PowerPoint presentations, case-based discussions, and interactive Q&A. Pre- and post-tests were delivered remotely via emailed PPT files with structured answer sheets returned by email. |
| **Duration (When and how much)** | Single full-day workshop, approximately 7.5 hours in total, delivered across morning, mid-morning, and afternoon sessions. The post-test was administered 4 weeks after the workshop. |
| **Content (What)** | Overview of dementia syndromes and clinical features; standard brain MRI protocols for dementia; introduction and application of visual rating scales (e.g., MTA, posterior atrophy, global cortical atrophy, Fazekas); signature structural MRI patterns of common and atypical dementias (Alzheimer’s disease, vascular dementia, frontotemporal dementia, dementia with Lewy bodies, multisystem atrophy, PSP, corticobasal degeneration, Creutzfeldt-Jakob disease, Huntington’s disease); case-based review of representative MRI cases. |
| **Materials (What-materials)** | PowerPoint slides summarising didactic content; anonymised high-resolution brain MRI cases in PPT format; structured answer sheets for theoretical and image interpretation tests; printed or projected copies of visual rating scale examples (if applicable). |
| **Procedures (How – sequence)** | (1) Baseline pre-training assessments (Pre-Test I: theoretical knowledge; Pre-Test II: image interpretation) administered remotely via email. (2) One-day in-person workshop with didactic sessions and interactive case discussions. (3) Four weeks later, post-training assessments (same format and cases as pre-tests) administered remotely via email. |
| **Tailoring / Individualisation** | The workshop was delivered as a standardised group program; content was not individually tailored, but facilitators encouraged questions and adapted case discussions to participants’ level of experience. |
| **Fidelity (Planned)** | A predefined curriculum and slide deck were used to ensure consistent delivery across all participants. The same trainers delivered all sessions. |
| **Fidelity (Actual)** | All planned teaching components were delivered as intended. Twenty-nine of 31 participants (93.5%) completed both pre- and post-training assessments |
