# Supplementary material for: A rapid educational intervention to enhance brain MRI interpretation skills of radiology professionals for dementia diagnosis in Uganda: a pre- and post-intervention study
Source: BMC Med Educ. 2026 Mar 12;26:642. doi: 10.1186/s12909-026-08997-z (PMC13094059; doi:10.1186/s12909-026-08997-z)
Supplement: Supplementary file 2 — Supplementary Material 2. [file 12909_2026_8997_MOESM2_ESM.docx]

# Supplementary file 2: STROBE Checklist for the training intervention

Reporting of this study followed the STROBE (Strengthening the Reporting of Observational Studies in Epidemiology) guidelines.

| **STROBE item** | **Recommendation (summary)** | **How it was addressed in this study** |
| --- | --- | --- |
| **1(a)** | Indicate the study design in the title. | The title describes the study as a pre- and post-intervention study. |
| **1(b)** | Give a clear and balanced summary of what was done and what was found in the abstract. | The abstract states the background, objective, methods, main results, and conclusion of the training intervention. |
| **2** | Explain the scientific background and rationale. | The introduction explains the burden of dementia, limited MRI use, and the gap in radiologist training in Uganda, giving the reason for the workshop. |
| **3** | State specific objectives and any hypotheses. | The objective is clearly stated: to assess the effect of a brain MRI dementia training workshop on theoretical knowledge and image-interpretation skills of radiologists and residents. |
| **4** | Present key elements of the study design early in the paper. | The methods describe a single-group pre- and post-test educational study, with participants taking tests before and after the workshop. |
| **5** | Describe the setting, locations, and relevant dates. | The study was done in the Department of Radiology at Makerere College of Health Sciences. The timing of the workshop and the 4-week post-test is described. |
| **6(a)** | Give eligibility criteria and how participants were selected. | Participants were radiologists and radiology residents who were actively reporting brain MRI within the last 3 months and who gave written informed consent. All eligible and available individuals were invited. |
| **6(b)** | For matched studies, give matching criteria and numbers. | Not applicable. Matching was not used. |
| **7** | Clearly define all outcomes, exposures, and other variables. | Primary outcomes were theoretical knowledge score and image-interpretation score. Other variables included sex, age group, cadre, and MRI experience. These are defined in the methods and tables. |
| **8** | For each variable, describe data sources and measurement methods. | Scores came from structured written tests: a multiple-choice theory test and image-interpretation cases scored with a predefined marking guide. All participants used the same tests and scoring method at both time points. |
| **9** | Describe efforts to address potential sources of bias. | The study used anonymised scripts, a standard marking scheme, and double scoring by senior radiologists with consensus to reduce observer bias. The discussion notes possible bias from unsupervised remote testing and a small sample size. |
| **10** | Explain how the study size was arrived at. | All eligible radiologists and residents who could attend the workshop were included (n=31). The study is presented as a pilot, and no formal sample size calculation was done. |
| **11** | Explain how quantitative variables were handled in the analyses. | Scores were treated as continuous and summarised using medians and IQRs. Age and MRI experience were grouped (<35 vs 35 years and <3 vs 3 years). Change scores were calculated as post-test minus pre-test. |
| **12(a)** | Describe all statistical methods. | The analysis used non-parametric tests. Wilcoxon signed-rank test was used for within-group changes, and Mann-Whitney U test for between-group comparisons. Normality was checked before choosing tests. |
| **12(b)** | Describe methods used to examine subgroups and interactions. | Subgroup analyses by cadre, MRI experience, age group, and sex were planned. Pre- and post-scores and change scores were compared across these groups using the same non-parametric tests. No interaction models were used. |
| **12(c)** | Explain how missing data were addressed. | Only participants with both pre- and post-test data (n=29) were included in paired analyses. This is stated in the results and table notes. |
| **12(d)** | For cohort or case-control studies, explain how loss to follow-up or matching was handled. | Not a classical cohort or case-control study. The small loss between baseline (31) and paired analysis (29) is reported, and analyses are based on complete cases. |
| **12(e)** | Describe any sensitivity analyses. | No formal sensitivity analyses were carried out. This is not applicable for this study. |
| **13(a)** | Report numbers of individuals at each stage of the study. | The results state that 31 participants completed the baseline assessment and workshop, and 29 completed both pre- and post-tests and were analysed as pairs. |
| **13(b)** | Give reasons for non-participation at each stage. | Two participants did not return their post-test within the stipulated time and did not respond to email reminders, so their data were excluded from the paired analyses. |
| **13(c)** | Consider using a flow diagram. | A separate flow diagram was not included. Participant flow is described in the text. |
| **14(a)** | Give characteristics of study participants and information on key variables. | Table 3 summarises age, sex, cadre, and MRI experience. The text describes that slightly more than half were female and that just over half were radiologists. |
| **14(b)** | Indicate the number of participants with missing data for each key variable. | There was no item-level missing data reported for key variables. Only 2 participants lacked post-test data. |
| **14(c)** | For cohort studies, summarise follow-up time. | Not a long-term cohort. The follow-up period is limited to a single post-test at 4 weeks, which is stated in the methods. |
| **15** | Report outcome data for each main outcome. | Pre- and post-training theoretical and image-interpretation scores are reported in the text, figures, and tables as medians with IQRs, along with p-values for change. |
| **16(a)** | Give unadjusted and, if applicable, adjusted estimates and their precision. | Unadjusted estimates (medians, IQRs, and p-values) are reported for total and subgroup scores. No adjusted analyses were done because of the small sample and pilot nature of the study. |
| **16(b)** | Report category boundaries when continuous variables are categorised. | Age and MRI experience group cut-offs (<35 vs 35 years; <3 vs 3 years) are reported in the methods and in table labels. |
| **16(c)** | If relevant, translate estimates into absolute risk or similar measures. | Not applicable. The study focuses on test scores, not risk estimates. |
| **17** | Report other analyses, such as subgroup and sensitivity analyses. | Subgroup comparisons by sex, cadre, age group, and MRI experience are reported in tables. No sensitivity analyses were done. |
| **18** | Summarise key results with reference to study objectives. | The discussion and conclusion restate that the workshop led to marked improvements in knowledge and image-interpretation skills, especially among less experienced readers, linking back to the main objective. |
| **19** | Discuss limitations, considering potential bias and imprecision. | The discussion highlights small sample size, no control group, possible contamination or help during remote tests, and short follow-up. The likely effect of these issues on the findings is discussed. |
| **20** | Give a cautious interpretation of the results, considering objectives, limitations, and other evidence. | The authors interpret the results carefully, relate them to other education and dementia imaging studies, and avoid overclaiming. They describe the workshop as a promising but early step. |
| **21** | Discuss the generalisability of the study results. | The discussion notes that findings are most relevant to similar LMIC training and academic hospitals and that external validity is limited by the single-country, single-department setting. |
| **22** | Give the source of funding and the role of funders. | National Institute of Neurological Disorders and Stroke, grant number D43TW010132; The funding section lists the supporting grant and states that the funder had no role in study design, data collection, analysis, or the decision to publish. |
